# A Novel Pyrazole Exhibits Potent Anticancer Cytotoxicity via Apoptosis, Cell Cycle Arrest, and the Inhibition of Tubulin Polymerization in Triple-Negative Breast Cancer Cells

**DOI:** 10.3390/cells13141225

**Published:** 2024-07-20

**Authors:** Edgar A. Borrego, Cristina D. Guerena, Austre Y. Schiaffino Bustamante, Denisse A. Gutierrez, Carlos A. Valenzuela, Ana P. Betancourt, Armando Varela-Ramirez, Renato J. Aguilera

**Affiliations:** 1 The Border Biomedical Research Center, The University of Texas El Paso, El Paso, TX 79968, USA; c.guerena7@gmail.com (C.D.G.); aschiaf@emory.edu (A.Y.S.B.); dagutierrez5@utep.edu (D.A.G.); car_los_alejandro@hotmail.com (C.A.V.); abetancourt@utep.edu (A.P.B.); avarela2@utep.edu (A.V.-R.); 2 Department of Biological Sciences, The University of Texas El Paso, El Paso, TX 79968, USA

**Keywords:** anticancer, drug screening, apoptosis, cell cycle arrest, transcriptome analysis, tubulin inhibition

## Abstract

In this study, we screened a chemical library to find potent anticancer compounds that are less cytotoxic to non-cancerous cells. This study revealed that pyrazole PTA-1 is a potent anticancer compound. Additionally, we sought to elucidate its mechanism of action (MOA) in triple-negative breast cancer cells. Cytotoxicity was analyzed with the differential nuclear staining assay (DNS). Additional secondary assays were performed to determine the MOA of the compound. The potential MOA of PTA-1 was assessed using whole RNA sequencing, Connectivity Map (CMap) analysis, in silico docking, confocal microscopy, and biochemical assays. PTA-1 is cytotoxic at a low micromolar range in 17 human cancer cell lines, demonstrating less cytotoxicity to non-cancerous human cells, indicating a favorable selective cytotoxicity index (SCI) for the killing of cancer cells. PTA-1 induced phosphatidylserine externalization, caspase-3/7 activation, and DNA fragmentation in triple-negative breast MDA-MB-231 cells, indicating that it induces apoptosis. Additionally, PTA-1 arrests cells in the S and G2/M phases. Furthermore, gene expression analysis revealed that PTA-1 altered the expression of 730 genes at 24 h (198 upregulated and 532 downregulated). A comparison of these gene signatures with those within CMap indicated a profile similar to that of tubulin inhibitors. Subsequent studies revealed that PTA-1 disrupts microtubule organization and inhibits tubulin polymerization. Our results suggest that PTA-1 is a potent drug with cytotoxicity to various cancer cells, induces apoptosis and cell cycle arrest, and inhibits tubulin polymerization, indicating that PTA-1 is an attractive drug for future clinical cancer treatment.

## 1. Introduction

Cancer is referred to as any disease that is characterized by abnormal and uncontrolled cell growth [1,2]. Normal cells become cancer cells when they stop responding properly to signals that control cellular homeostasis. After cancer has initiated, cells will divide indefinitely, metastasizing, invading normal tissue and organs, and killing the host if it is left untreated [3]. Cancer is the second leading cause of death worldwide [4,5], and it accounts for one in six deaths [6]. In 2020, 19.3 million new cases of cancer appeared, and about 10 million cancer deaths were reported worldwide [7]. In the USA, it is estimated that there will be 2,001,140 new cancer cases and around 611,720 new deaths by 2024 [8]. By 2040, 28.4 million cancer cases are projected, almost 50% more than in 2020 [7]. Thus, cancer represents a global health problem. Although cancer continues to be a leading cause of death, the number of survivors in the United States has increased, which is due to improvements in early detection and treatment [9]. Despite the progress in the development of cancer treatments, chemotherapy remains the standard treatment for many cancers. Although chemotherapy has improved the survival rates and is effective in killing cancer cells, it can also kill normal cells, leading to life-threatening side effects [10,11]. Thus, chemotherapeutic agents with high cytotoxicity and selectivity towards cancer are needed. 

Microtubules are polymers composed of tubulin proteins that maintain the cellular shape (cytoskeleton) and are essential for mitotic spindle formation and chromosome segregation during mitosis [12]. Compounds targeting tubulin can prevent microtubule polymerization or block their dynamic function, leading to early mitosis arrest, proliferation blockade, and, in some cases, cell death [10,13]. In fact, tubulin represents one of the most effective targets for antitumor drugs [14,15]. Tumor cells use many mechanisms to produce resistance that involve the use of efflux pumps and the presence of mutations and/or the expression of different tubulin isotypes. Hence, it is highly important to increase the repertoire of functional anticancer drugs [13]. 

In this study, a novel pyrazole derivative, 2-{4-[4-methoxy-3-(trifluoromethyl)phenyl]-1H-pyrazol-1-yl}-N-(2-methyl-2H-1,2,3-triazol-4-yl) acetamide (known as PTA-1), which has potent cytotoxicity against a panel of different cancer cell lines, is described. Our results indicate that PTA-1 induces apoptosis at a low micromolar concentration in MDA-MB-231 cells, as measured by the externalization of phosphatidylserine (PS), the activation of caspase-3/7, and the detection of DNA fragmentation. Furthermore, PTA-1 alters the cell cycle profile, causing arrest in the S and G2/M phases, producing a more prominent effect in the G2/M phase. Moreover, as shown by fluorescent microscopy images and tubulin polymerization assays, PTA-1 disturbs microtubule organization and inhibits tubulin polymerization, as determined by a polymerization inhibition assay. Since tubulin polymerization inhibition has been proven to be a successful strategy in cancer treatment, PTA-1 exhibits the characteristics of a chemotherapeutic anticancer agent.

## 2. Materials and Methods

### 2.1. Chemicals

Compounds for the primary screening were acquired from the Chembridge DIVERset library (Chembridge Corp, San Diego, CA, USA) and were already diluted in dimethyl sulfoxide (DMSO) at a concentration of 10 mM. The experimental compound PTA-1 (2-{4-[4-methoxy-3-(trifluoromethyl)phenyl]-1H-pyrazol-1-yl}-N-(2-methyl-2H-1,2,3-triazol-4-yl) acetamide), with Chembridge reference ID #31999318, was acquired for additional tests. Etoposide (# 34120525MG), Paclitaxel (#AC328420050), and vinblastine (#AAJ63598MA) were acquired from Fisher Scientific. All compounds were obtained lyophilized and later dissolved in DMSO.

### 2.2. Cell Culture

All cell lines used in this study were purchased from the ATCC and were previously established from human subjects. The following adherent breast-cancer-derived cell lines were used: MDA-MB-231, MCF-7, HCC70, and T47D. Additionally, two cell lines derived from patients with colon cancer, COLO 205 and HT29, as well as lung cancer A549, liver cancer HepG2, melanoma A375, pancreatic PANC-1, and prostate cancer PC3 cell lines, were used. In addition, one non-cancerous cell line derived from breast tissue, MCF-10A, was included in this study. Moreover, the hematological cancer cells Ramos (Burkitt’s lymphoma), HL-60 (acute promyelocytic leukemia), CCRF-CEM (acute lymphoblastic leukemia), Nalm6 (acute lymphoblastic leukemia), Jurkat (acute T cell leukemia), KCL22 (chronic myeloid leukemia), and K562 (chronic myeloid leukemia) were also used. Cells were grown using different media. RPMI medium supplemented with 10% FBS was used for leukemias and lymphomas, except for HL-60, which was supplemented with 20% FBS. The HCC70, PC3, and COLO 205 cell lines were also grown using RPMI prepared with 10% FBS. For MDA-MB-231, HepG2, PANC-1, A375, A549, and MCF-7, DMEM with 10% FBS was used. The MCF-10A cell line was grown using DMEM F/12 media containing 10% FBS, 10 µg/mL of insulin, 20 ng/mL of epidermal growth factor (EGF), 0.5 µg/mL of hydrocortisone, and 10 µg/mL of insulin. HT-29 cells were grown in McCoy’s media supplemented with 10% FBS. The following antibiotics were added to all media: 100 U/mL of penicillin and 100 µg/mL of streptomycin. All cell lines were cultured in a humidified environment with 5% CO_2_ at 37 °C and monitored daily to avoid 100% cellular confluency and the acidification of the culture media. To avoid 100% confluency, the media were replaced every 2–3 days, and cells that were not needed were discarded. For the hematological cancer cells, viability was measured via flow cytometry using propidium iodide (PI) [16] at a final concentration of 5 µg/mL to identify dead cells before every experiment, and only cells with 95% or more viability were used.

### 2.3. Differential Nuclear Staining (DNS) Assay

To determine the cytotoxic activity of PTA-1 in different cancerous and non-cancerous cell lines, the differential nuclear staining (DNS) assay was used [17]. To perform the DNS assay, 1 × 10^4^ cells were seeded in 96-well plates at a volume of 100 μL of media per well, and plates were placed under optimal cell culture conditions overnight. In the primary screening, cells were treated with 5 μM of each compound. After identifying the PTA-1 compound, a concentration gradient of PTA-1 was used for the secondary screening. DMSO at 1% *v*/*v* was used as a vehicle control, and untreated cells were used as a negative control. H_2_O_2_ was added to the cells to induce cell death as a positive control at 1.6 millimolar (mM). An average was calculated from three independent measurements to calculate the cytotoxicity for each concentration. Six independent measurements were used for DMSO and untreated cells. After treatments were added, cells were left for a period of 24, 48, or 72 h, and, 2 h before the incubation period, a mix containing Hoechst 33342 and PI (5 µg/mL) in phosphate-buffered saline (PBS) was added to each well and the plates were read using a GE Healthcare IN Cell Analyzer 2000 system. Four adjacent images (2 × 2) per well were captured with a 10× objective for each dye and were analyzed with the IN Cell Analyzer Workstation 3.2 software (GE Healthcare, Pittsburgh, PA, USA). 

### 2.4. Cytotoxic Concentration 50 (CC_50_) and Selective Cytotoxicity Index (SCI) Calculation

The CC_50_ was calculated by extrapolating two concentrations, one that killed more than 50% of the population and one that killed less than 50% [18]. The extrapolation was accomplished by using an online tool (https://www.johndcook.com/interpolator.html accessed on 2 June 2022). At least three replicates were used for each concentration. The cytotoxicity of each treatment was normalized to the DMSO control, as previously described [19]. In addition, to determine if a compound had selectivity towards cancer preferentially over non-cancerous cell lines, the selective cytotoxic index (SCI) was calculated. The SCI is calculated by dividing the CC_50_ value of the non-cancerous cell line (MCF-10A) by the CC_50_ value of the cancer cell lines. A value > 1 indicates that the compound kills cancer cells better than non-cancerous cells [20].

### 2.5. Annexin V-FITC and Propidium Iodide Assay

Phosphatidylserine externalization was determined using the Annexin V-FITC and Propidium Iodide Kit (Beckman Coulter, Miami, FL, USA). MDA-MB-231 cells were seeded in 24-well plates at a density of 1 × 10^5^ cells in a volume of 1000 µL of complete growth media per well, and plates were placed overnight under optimal cell culture conditions. The next day, cells were treated with PTA-1 at 10 and 20 μM, 0.1% *v*/*v* of DMSO, and 1.6 mM of H_2_O_2_ as vehicle and positive controls, respectively. Untreated cells were used as a negative control. After 24 h of treatment, cells were collected in flow cytometry tubes, which were centrifuged for 5 min at 1200 rpm and 4 °C. The supernatant was discarded, and cells were resuspended in 100 µL of a combination that contained 1× binding buffer (BB), 1 µL of Annexin V-FITC, and 2.5 µL of PI. Cells were then left for 15 min on ice in the dark, as previously described [21]. Finally, 300 µL of 1× BB was added to each tube, and samples were examined via flow cytometry. Approximately 1 × 10^4^ cells/events were read per sample. To capture the fluorescence signal emitted by FITC, an FL1 detector was used, whereas, for PI, an FL2 detector was used [22]. FITC-positive cells were considered early apoptotic cells, whereas PI-positive cells were considered necrotic cells. The double-positive FITC and PI cells represented late apoptosis.

### 2.6. Caspase-3/7 Activation Assay

Live-cell detection was achieved via flow cytometry examining caspase-3/7 activity with the fluorogenic reagent NucView 488 caspase-3/7 substrate (Biotium, Hayward, CA, USA). For this assay, MDA-MB-231 cells were also seeded at a density of 1 × 10^5^ cells in 1000 µL of complete media per well in 24-well plates. Then, the plates were left under optimal cell growth conditions overnight and treated with PTA-1 the following day for 7 h. The same controls were employed as aforementioned. DMSO was used as a vehicle (solvent) control, and untreated cells were used as the negative control. As a positive caspase-3/7-activating control, cells were treated with H_2_O_2_. After treatment, cells were harvested in flow cytometry tubes to be centrifuged for 5 min at 1200 rpm. The supernatant was discarded, and 200 µL of PBS containing 5 µL NucView 488 Caspase-3/7 substrate (5 µM final concentration) was added to each tube; they were then incubated in the dark for 30 min at room temperature. Subsequently, cells were washed with PBS and resuspended with 400 µL of PBS and then immediately analyzed through flow cytometry using the FL1 detector [23].

### 2.7. Cell Cycle Analysis

To analyze whether PTA-1 altered the cell cycle, 1 × 10^5^ cells were seeded in 24-well plates with a volume of 1000 µL of media per well and were placed overnight under cell culture conditions. The following day, cells were treated with 1.25 µM, 2.5 µM, and 5 μM of PTA-1 for 72 h. After incubation, the media were collected, and cells were detached and collected in flow cytometry tubes. Afterward, tube-containing cells were centrifuged for 5 min at 1200 rpm to subsequently discard the supernatant. Next, cells were resuspended with a mixture containing 200 µL of NIM-DAPI and 200 µL of PBS and were read via flow cytometry. Cells that were stained with DAPI in complex with DNA were read with an FL9 detector. At least 20,000 events were obtained per sample [24] and were analyzed with the Kaluza 1.3 Software (Beckman Coulter, Brea, CA, USA). The following four subpopulations were identified: sub-G0/G1, G0/G1, S, and G2/M.

### 2.8. Transcriptome Analysis

The transcriptome analysis was accomplished by seeding 1 × 10^6^ MDA-MB-231 cells at a volume of 5 mL in T-25 flasks and culturing them overnight. The next day, cells were treated for 24 h with 0.1% DMSO *v*/*v* and 20 µM of the PTA-1 compound and placed in the incubator. It is important to note that the CC_50_ of PTA-1 on MDA-MB-231 was determined to be 10 μM at 24 h and twice this concentration was used to ensure a strong induction in cell death. To avoid DMSO solvent effects on the expression analyses, the DMSO concentration was reduced 10-fold to 0.1% in all treatments. After incubation, the media were collected in 15 mL conical tubes, and cells were detached by adding 1000 µL of 1× trypsin to each flask, and they were placed back into incubation. After this step, 1000 µL of complete medium was added to each flask, and cells were collected in their respective tubes, which were centrifuged for 5 min at 1200 rpm. The supernatant was disposed of, and cells were then washed with 1 mL of warm PBS to remove leftover media, followed by centrifugation and discarding the supernatant. After this, the RNA was extracted using the RNeasy Mini Kit (Qiagen, Germantown, MD, USA), following the manufacturer’s instructions. A NanoDrop spectrophotometer measured the RNA purity and quantity. The following steps were performed as previously reported [25]. RNA samples with A260/A280 readings between 1.8 and 2.1 were considered for RNA sequencing. To perform RNA sequencing, the RNA concentrations and integrities were measured with the RNA BR Assay Kit for Qubit 3.0 (Invitrogen, Waltham, MA, USA) and RNA ScreenTape in a 4200 TapeStation (Agilent, Santa Clara, CA, USA), respectively. Next, TruSeq Stranded mRNA by Illumina was used for library preparation. After the libraries were pooled and diluted to the loading concentration, they were sequenced using a NextSeq 1000/2000 P2 reagent with 200 cycles (Illumina, San Diego, CA, USA) in a NextSeq 2000 system (Illumina San Diego, CA, USA), following the manufacturer’s instructions. For the RNAseq data analysis, the raw reads were trimmed using Trimmomatic (v0.38) [26], aligned, and quantified within RSEM [27] using Bowtie2 [28]. Differential analysis was performed using DESeq2 [29]. A list of significant genes was extracted using a p-adjusted cutoff of less than 0.05 and fold change greater than 2. Fold changes were determined by the comparison of PTA-1-treated vs. solvent-control-treated samples [25].

### 2.9. Connectivity Map (CMap) Analysis

To analyze the similarity of the gene expression profile produced by PTA-1 to those of other known drugs, MDA-MB-231 cells were treated with PTA-1 treatment for 24 h and the differentially expressed genes (DEGs) were compared to the drug-induced patterns within the Connectivity Map (CMap) database. The CMap database contains the gene expression profiles of small molecules tested in multiple cell lines [30,31]. The website https://clue.io (accessed on 16 August 2022) was used for the tool query with the parameters Gen expression (L1000), an individual query, and 1.0. After 24 h of PTA-1 treatment, 150 upregulated and 150 downregulated DEGs were compared; these were affected 2-fold or more as determined by comparison to the solvent control. The perturbational class member option was selected to sort the most similar compounds based on the tau score, and the top 10 hit compounds were chosen.

### 2.10. Computational Docking

The Schrödinger software release 2022-2 was used to accomplish in silico molecular docking as previously described [23,32]. For this analysis, compound PTA-1 and the positive control benzylidene derivative of 9(10H)-anthracenone (TPI-1 or 89U) were examined using the software’s Ligprep interface with OPLS3 at pH 7 ± 2 utilizing Epik. Other included options were set as default on the software. The structure of tubulin was obtained from the Protein Databank (PBD:5XLZ) (https://www.rcsb.org/structure/5XLZ accessed on 24 September 2022) and was prepared as previously described [33]. The structure of tubulin was previously reported to be crystallized in a complex with 10-[(4-methoxy-3-oxidanyl-phenyl)methylidene]anthracen-9-one, also known as 89U and TPI-1. In silico docking was performed on the same site where 89U was bound. The sitemap tool in the software was used to define the receptor grid generation. Molecular docking and molecular mechanics were performed using the glide and prime tools on Maestro 13.0, respectively.

### 2.11. Cytoskeleton Analysis with Fluorescent Microscopy

Fluorescent microscopy was accomplished by seeding 2 × 10^3^ cells in 100 µL of media per well in 96-well plates (BD Falcon, 353216), which were left overnight under optimal cell culture conditions. The following day, cells were treated for 4 h with 20 µM of PTA-1, 1 µM of Paclitaxel (PTX), and DMSO (1% *v*/*v*) as a solvent control. Untreated cells were used as a negative control. Cells were then fixed by adding 100 µL of 8% formaldehyde to each well and were left for 20 min at room temperature (RT). Next, the formaldehyde and media were removed, and the wells were washed three times with 200 µL of a combination of PBS and 0.1% Tween 20 detergent for 10 min at RT. These washes allowed the removal of any leftover media and formaldehyde and cell permeabilization. After three washes, a blocking solution containing Tris-buffered saline with 0.1% Tween 20 and 5% bovine serum albumin (BSA) was added to each well, and the plates were placed for an incubation period of 1 h on a rocking platform at RT. Following incubation, the blocking solution was removed, and 50 µL of a mixture containing 0.1% Tween 20 detergent in PBS, DAPI at a final concentration of 5 µg/mL, phalloidin conjugated to Alexa Fluor 568 at 0.165 µM, and an anti-tubulin conjugated to Alexa Fluor-488 monoclonal antibody was added to each well to stain the cells. Plates were incubated overnight on a rocking platform at 4 °C. After incubation, the mixture was recovered from each well, and cells were washed three times, as described above. High-quality images were captured using an LSM-700 laser scanning confocal microscope (Zeiss) with an EC Plan-Neofluar 40×/1.30 oil DIC objective. Image acquisition and analysis were accomplished with the Zen 2009 6.0 software (Zeiss, Oberkochen, Germany).

### 2.12. Tubulin Polymerization Assay

To test the potential activity of PTA-1 as a tubulin polymerization inhibitor, a tubulin polymerization assay kit (cat. # BK006p) purchased from Cytoskeleton Inc. (Denver, CO, USA) was used. The assay was performed by following the manufacturer’s protocol. Before running the assay, a general tubulin buffer (GT) containing 80 mM of PIPES buffer (piperazine-N, N′-bis(2-ethanesulfonic acid)), 2 mM of magnesium chloride (Mg_2_CL), and 0.5 mM of ethylene glycol-bis(b-amino-ethyl ether) N, N, N′, N′-tetra-acetic acid (EGTA) was reconstituted with 10 mL of sterile distilled water. GTP was also reconstituted with sterile water at a concentration of 100 mM. Then, 10 mg of lyophilized tubulin protein was reconstituted with 10 µL of GTP at 100 mM and 1.1 mL of general tubulin buffer (GT). GTP stocks were kept at −80 °C, and tubulin was frozen using liquid nitrogen and kept at −80 °C. Before starting the assay, the sample plate was pre-warmed at 37 °C for 30 min. Then, the tubulin polymerization buffer (TP) was prepared by mixing general tubulin buffer, tubulin glycerol buffer, and GTP at a final concentration of 15% glycerol and 1 mM, respectively. Next, 10 µL of general tubulin buffer was added to each well. Treatments were then added to each corresponding well, including PTA-1 at 10 µM, 0.9% of DMSO as a vehicle control, 10 µM of PTX as a tubulin stabilizer, and 3 µM of vinblastine as a positive control. After treatment, tubulin was diluted and mixed with ice-cold TP for a final concentration of 3 mg/mL and then 100 µL of the solution was rapidly added to all wells with the treatments. Plates were placed in the spectraMax spectrophotometer (Molecular Devices, San Jose, CA, USA) at 37 °C and read for 1 h, taking reads at a 340 nm wavelength every minute. An automatic mix was performed before the first read.

## 3. Results

### 3.1. Drug Screening Identified a Novel Pyrazole Derivative with Potent Cytotoxicity against a Panel of Different Cell Lines

**Figure 1:**
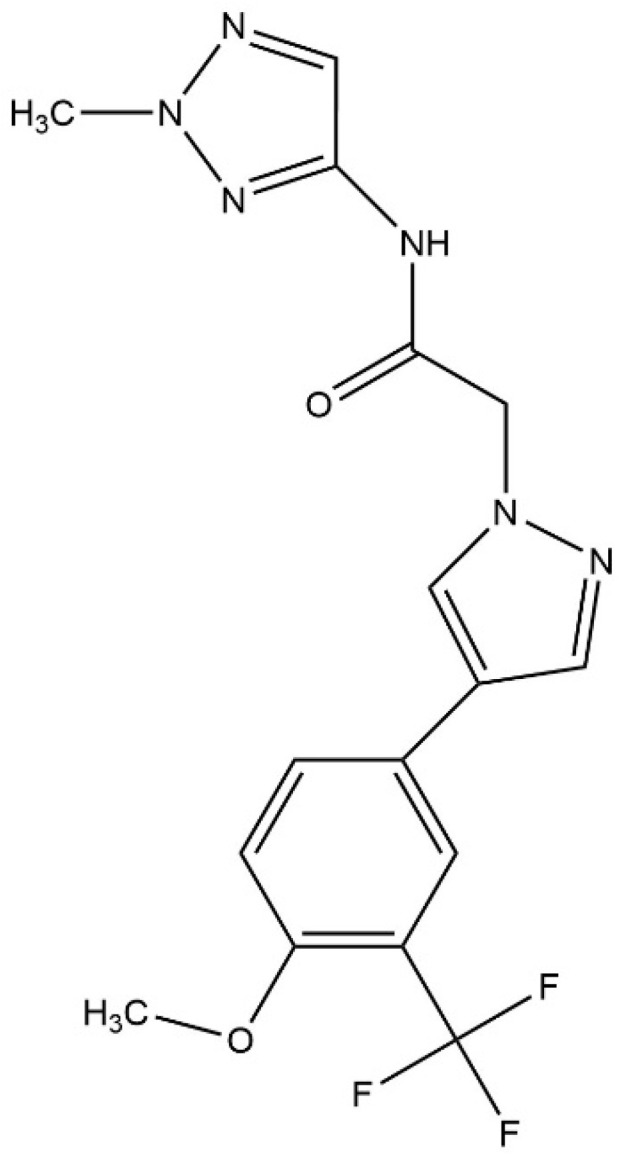
PTA-1 chemical structure. The chemical structure of 2-{4-[4-methoxy-3-(trifluoromethyl)phenyl]-1H-pyrazol-1-yl}-N-(2-methyl-2H-1,2,3-triazol-4-yl) acetamide, also known as PTA-1.

An initial screening of 5600 compounds from the ChemBridge DIVERset chemical library was performed on the cell line CCRF-CEM to identify potent cytotoxic molecules. This first screen detected 10 small molecules with potent cytotoxic activity, and these compounds were subsequently retested to verify their activity. Of the identified compounds, a novel pyrazole, PTA-1 (Figure 1), was identified to be the most cytotoxic; for this reason, PTA-1 was tested in a panel of different cancer and non-cancerous cell lines (Table 1). The activity of PTA-1 was tested in leukemias and lymphoma cells after 48 h of incubation, whereas cells derived from solid tumors and the non-cancerous MCF-10A cells were treated for 72 h (Table 1), since the latter grew at a much slower pace. The most sensitive cell line to PTA-1 among the leukemias and lymphomas was Jurkat, with a CC_50_ value of 0.32 µM. In contrast, the most sensitive cell line within the adherent solid tumor cell lines was the lung A549 adenocarcinoma cell line, with a CC_50_ value of 0.17 µM. The least sensitive cancer cell line was MDA-MB-231, with a PTA-1 CC_50_ value of 0.93 µM. In addition, the non-cancerous cell line, MCF-10A, was less sensitive to the toxic activity of PTA-1, displaying a CC_50_ of 4.40 µM (Table 1). The selective cytotoxicity index (SCI) was calculated to determine the preferential cytotoxicity towards cancer cells. A value greater than one indicates that a compound preferentially kills cancer cells. All SCI values were greater than one, meaning that PTA-1 has selectivity for cancer cells (Table 1). These results suggest that PTA-1 has potent cytotoxicity against cancer cells. Thus, PTA-1 is a promising candidate as an anticancer drug. To further characterize PTA-1, the MDA-MB-231 breast cancer cell line was used to perform additional confirmatory experiments.

### 3.2. PTA-1 Induces Apoptosis as Measured by Phosphatidylserine Externalization and Caspase-3/7 Activation

**Figure 2:**
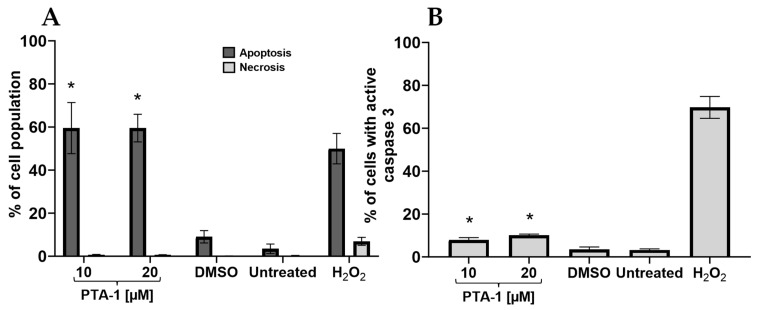
PTA-1 induces apoptosis. The externalization of PS and the activation of caspase-3/7 indicates that PTA-1 induces apoptosis. (**A**) Treatment of MDA-MB-231 cells with PTA-1 at concentrations of 10 and 20 μM for 24 h triggers apoptosis, as PS externalization shows. (**B**) The fluorogenic reagent NucView 488 caspase-3/7 substrate was used to detect the activation of caspase-3/7 via flow cytometry. MDA-MB-231 cells treated with 10 and 20 μM of PTA-1 compound for 7 h significantly induced the activation of caspase-3/7, suggesting apoptosis as the cell death mechanism induced by PTA-1. * indicates *p*-value lower than 0.01. An average of 3 independent determinations is represented by each bar.

After identifying PTA-1, we examined the MOA of the compound. Other pyrazole derivatives have been demonstrated to induce apoptosis as the primary cell death mechanism [19,25]; therefore, the activity of PTA-1 has been tested as a potential inducer of apoptosis. The Annexin V assay measures the externalization of phosphatidylserine (PS), which is usually present in the inner sides of healthy cell membranes but is externalized when cells undergo apoptosis [34]. PTA-1 was used at 24 h CC_50_ (10 µM) and 2× CC_50_ (20 µM) concentrations, and, at both concentrations, PTA-1 induced the externalization of PS in 59.4% and 59.7% of MDA-MB-231 cells, respectively (Figure 2A). As expected, in untreated cells, just 3.4% of the cell population was positive for apoptosis, while the DMSO vehicle control induced 9% PS externalization. On the other hand, the H_2_O_2_ positive control induced PS externalization in 49.9% of the population (Figure 2A). The comparison between PTA-1 treatment and DMSO was significantly different (*p* ≤ 0.001002), indicating that PTA-1 does trigger a significant amount of PS externalization. In order to corroborate that PTA-1 induces apoptosis, the activation of caspase-3/7 was investigated. During the induction of apoptosis, caspase-3/7 is activated to cleave more than 500 substrates that initiate apoptosis [35]. Our data revealed that caspase-3/7 was indeed activated after 7 h of exposure to PTA-1 in MDA-MB-231 cells at both PTA-1 concentrations. A total of 7.51% of cells showed an increase in caspase-3/7 activation at 10 μM, whereas 9.91% was observed when 20 μM of PTA-1 was used (Figure 2B). In contrast, 3.9% of the cell population was positive for activated caspase-3/7 when exposing cells to the vehicle control, DMSO, at 0.1% *v*/*v* (Figure 2B). Furthermore, a significant difference between DMSO and the PTA-1 compound was observed (*p* ≤ 0.00421), indicating that caspase-3/7 was activated by PTA-1, confirming apoptosis as the main cell death mechanism.

### 3.3. PTA-1 Interferes with Cell Cycle Progression

**Figure 3:**
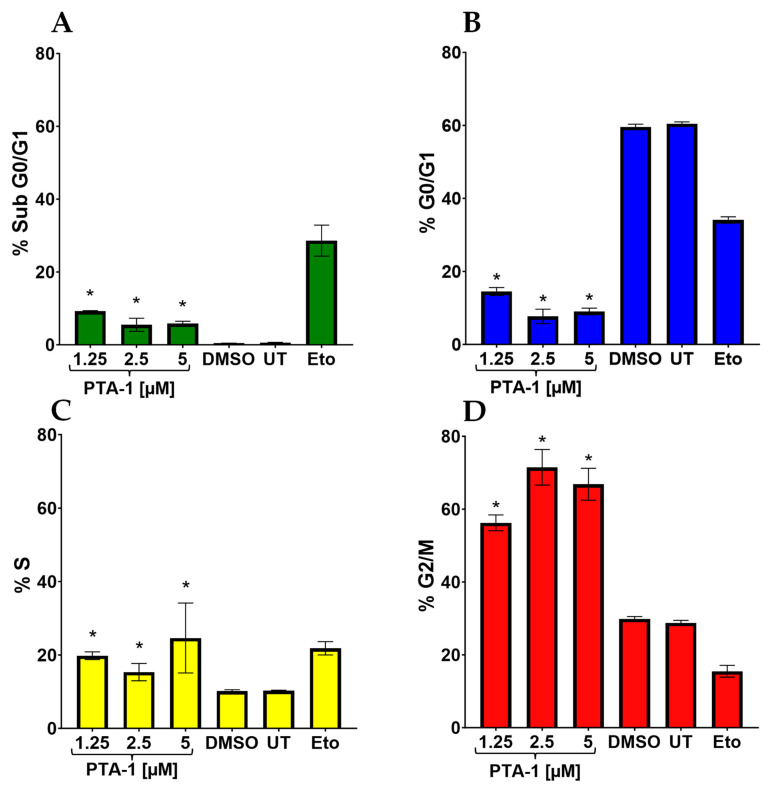
PTA-1 alters the cell cycle by arresting cells in the S and G2/M phases in MDA-MB-231 cells. Exposure of MDA-MB-231 cells to PTA-1 for 72 h causes the alteration of the cell cycle. (**A**) PTA-1 at 1.25 µM, 2.5 µM, and 5 µM causes significant DNA fragmentation, as measured by the hypodiploid cells that are present in the sub-G0/G1 subpopulations. (**B**) PTA-1 treatment did not arrest cells in the G0/G1 phase. However, fewer cells in this phase were seen after treatment with the three concentrations of PTA-1. MDA-MB-231 cells exposed to PTA-1 significantly arrested the cells in the S (**C**) and the G2/M phases (**D**); nonetheless, the effect was more noticeable in the G2/M phase. Asterisk (*) denotes the *p*-value being <0.05 as compared to DMSO.

Compounds that arrest the cell cycle are effective in stopping cell proliferation [36]. Therefore, we decided to assess the MDA-MB-231 cell cycle after treatment with PTA-1 to examine its influence on cell cycle progression. To avoid a high percentage of DNA fragmentation produced by PTA-1, we used half, a quarter, and an eighth (5 μM, 2.5 μM, and 1.25 μM) of its CC_50_ value on MDA-MB-231. Readings were taken after 72 h of exposure to the compound by flow cytometry. Since these analyses were performed after 72 h of incubation, lower concentrations of PTA-1 were added to reduce the levels of DNA degradation that would interfere with the detection of cell cycle arrest. A slight but significant increase was observed after PTA-1 treatment in the sub-G0-G1 phase (*p* ≤ 0.004048) when compared to the DMSO control (Figure 3A). Cells in the sub-G0/G1 phase indicate cells with fragmented DNA that underwent apoptosis. Moreover, when compared to DMSO and the untreated controls, an important increment was determined in the S and G2/M phases in all PTA-1 treatments (Figure 3C,D). Cells treated with increasing concentrations of PTA-1 arrested 19.8%, 15.34%, and 24.6% of the cells in the S phase (Figure 3C). A comparison with DMSO treatment indicated a significant increment in all treatments (*p* ≤ 0.029368). The effect of PTA-1 was more dramatic in the G2/M phase, with greater than 50% (from 53.25% to 71.5%) of cells that were arrested in this phase after treatment with the three concentrations (*p* ≤ 0.000281) (Figure 3D). Additionally, a decrease in cells going through the G0/G1 phase was seen after treatment with PTA-1 (Figure 3B), likely due to the increase seen in the other phases of the cell cycle. As anticipated, untreated cells and cells treated with DMSO progressed normally through all cell cycle phases (Figure 3A–D). Etoposide-treated cells produced a high percentage of apoptotic cells compared to the DMSO (Figure 3A) and arrested cells in the S phase (Figure 3C). Etoposide inhibits mitosis and stops cell proliferation in the S and G2/M phases [37]. However, we did not see an increase in the G2/M phase. In general, these data reveal that PTA-1 acts by arresting cells in the S and G2/M phases, with a more pronounced effect in the G2/M phase. Hence, these results indicate that PTA-1 treatment results in the accumulation of cells in the G2/M phase.

### 3.4. PTA-1-Treated MDA-MB-231 Cells Display a Transcriptome Profile Similar to Tubulin Polymerization Inhibitors

**Figure 4:**
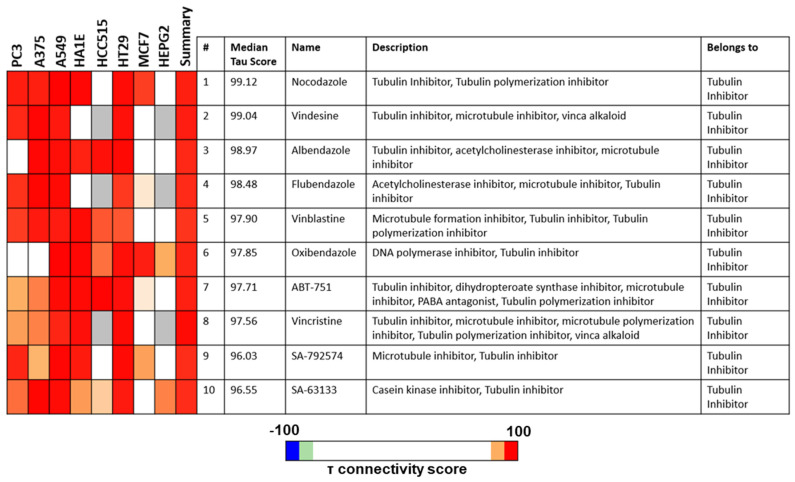
CMap analyses show that PTA-1 treatment results in a gene expression profile similar to that of tubulin inhibitors. MDA-MB-231 cells treated with PTA-1 for 24 h produced a gene expression profile similar to that of known tubulin inhibitors. Results are shown as a heat map in 8 cell lines. The most robust red color means higher positive connections. The top 10 hit compounds based on the tau median score were selected, and all top 10 compounds with gene expression signatures similar to PTA-1 belonged to tubulin inhibitors.

It has been previously shown that different inhibitors targeting the same protein produce similar gene expression responses [38,39]. To elucidate the mode of action of PTA-1, a global analysis of RNA transcription in MDA-MB-231 was performed after cells were treated with PTA-1 for 24 h. After comparison to the expression patterns of the DMSO control-treated cells, the fold effects were calculated. RNA sequencing after PTA-1 treatment resulted in the detection of 198 upregulated and 532 downregulated DEGs. After the detection of these DEGs, we sought to identify the mode of action of PTA-1 by comparing its gene expression signature to those produced by other small molecules whose mode of action has been determined. The Connectivity Map (CMap) contains the gene expression profiles of different cancer cell lines treated with various compounds [40]. Therefore, the gene expression signature of MDA-MB-231 cells treated with PTA-1 for 24 h was analyzed using this database. For this analysis, 150 upregulated (Supplementary Table S1) and 150 downregulated genes (Supplementary Table S2) were compared to the CMap database (300 DEGs is the input limit). To compare PTA-1 to the CMap database, the compounds were sorted according to the option of the perturbational class member, and the top ten compounds were selected based on the connectivity score (tau). A positive score indicates that there is similarity, and the magnitude of the score equals the magnitude of similarity [41]. Figure 4 shows the connections represented as a heat map that includes scores for eight cell lines. The strongest red color indicates stronger similarity to the perturbagen. After 24 h of PTA-1 treatment, the gene expression profile was found to be similar to the signatures produced by tubulin inhibitors (Figure 4). In fact, all top 10 compounds are known tubulin inhibitors. Thus, these data show that the gene expression profile produced in cells treated with PTA-1 is similar to that of known tubulin inhibitors, suggesting that PTA-1 could inhibit tubulin polymerization. 

### 3.5. Computational Docking Shows That PTA-1 Interacts with Tubulin

**Figure 5:**
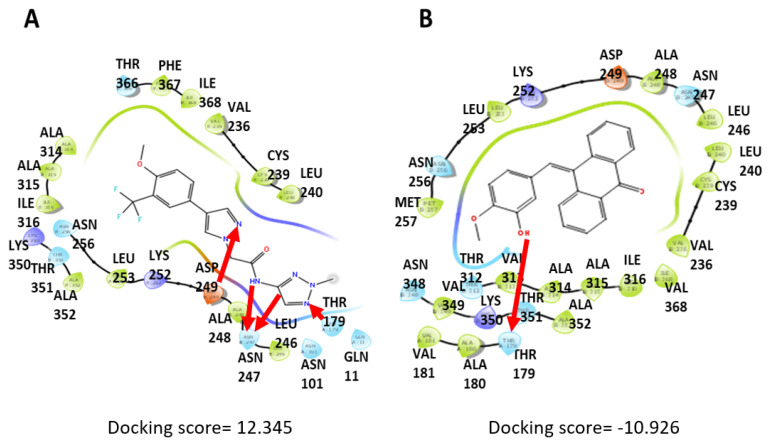
Schrödinger interaction diagrams of PTA-1 and U89 with tubulin. Molecular docking using the Schrödinger software shows the interactions between different molecules and tubulin, and the interactions with the best scores were selected. (**A**) Diagram shows the PTA-1 hydrogen bond with THR179, ASN247, and ASP249 (red arrows). This interaction resulted in a docking score of −12.345 and an MMGBSA value of −73.78 Kcal/Mol. (**B**) Compound 89U, used as a positive interaction control, displays a score of −10.926 and an MMGBSA value of −72.89 Kcal/Mol.

After determining that PTA-1 has a gene expression signature similar to well-known tubulin inhibitors, the possibility that PTA-1 could inhibit tubulin was explored. An in silico assay was performed to evaluate PTA-1’s interaction with tubulin, and the Schrödinger software release 2022-2 was used to accomplish molecular docking. The structure of tubulin was obtained from the Protein Databank (PBD:5XLZ), which was crystallized in a complex with 10-[(4-methoxy-3-oxidanyl-phenyl)methylidene]anthracen-9-one, also known as 89U or TPI1. After the docking simulations, the best interactions for each molecule were selected. According to the Schrodinger website, a Glidescore of −10 or lower represents a good binding score [42], and since the Glidescore and docking scores are similar [43], −10 was deemed a good binding score. As expected, the positive control, 89U, showed a good binding score of −10.9 (Figure 5B), and PTA-1 exhibited a docking score of −12.3 (Figure 5A), implying stronger binding than U89. In addition, PTA-1 showed a Molecular Mechanics with a Generalized Born and Surface Area (MMGBSA) value of −73.78 Kcal/Mol. In comparison, U89 had a value of −72.89 Kcal/Mol (Figure 5A,B), with a more negative value indicating stronger binding [44]. As shown in Figure 5, both PTA-1 and U89 form hydrogen bonds (indicated with red arrows) with THR179. Moreover, PTA-1 interacts via the pyrazole moiety with additional groups, forming hydrogen bonds with THR179, ASN247, and ASP249 (red arrows). Thus, our data suggest that PTA-1 exhibits stronger binding to tubulin than U89 in this in silico model.

### 3.6. PTA-1 Disturbs Microtubule Organization in MDA-MB-231 Cells

**Figure 6:**
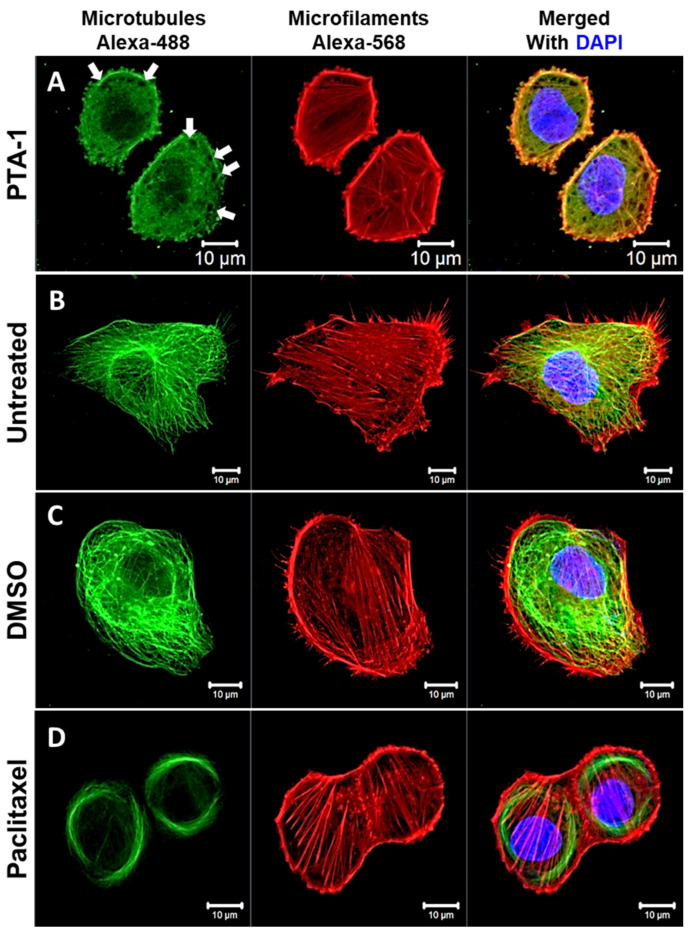
PTA-1 disturbs microtubule organization in MDA-MB-231 cells. Cells were stained with an anti-α-tubulin antibody conjugated to Alexa-488 (microtubules), phalloidin–Alexa-568 (microfilaments, polymerized actin), and DAPI (nucleus) and analyzed via confocal microscopy. (**A**) Four hours of PTA-1 treatment disrupted microtubule organization in MDA-MB231 cells. White arrows indicate empty regions where tubulin is absent. (**B**) Untreated, (**C**) DMSO (vehicle), and (**D**) Paclitaxel (microtubule-stabilizing agent) controls were included.

Previous research has demonstrated that microtubules are directly disrupted by tubulin inhibitors [45,46]. To test if PTA-1 produces an alteration in the microtubule organization, the cytoskeleton morphology was assessed in MDA-MB-231 cells treated with 20 µM of PTA-1 using confocal fluorescence microscopy. Control treatments included DMSO (vehicle), Paclitaxel, a microtubule-stabilizing agent, and untreated cells as a negative control. PTA-1 completely disrupted the tubular (filaments/fibers) structure of microtubules after treating MDA-MB-231 cells for 4 h (Figure 6). Interestingly, PTA-1 produced empty regions lacking tubulin, as shown in Figure 6 with white arrows. Moreover, PTA-1-treated cells were smaller and rounder than cells treated with DMSO and PTX and untreated cells. This shrinking/rounding effect and regions lacking tubulin (white arrows) were also seen with two additional cancer cell lines, the PANC-1 pancreatic and the A549 lung cancer cell lines (see Supplementary Figures S1 and S2). It is important to note that a similar effect on membrane disruption and cell rounding was also detected with the control MCF-10A cell line treated with PTA-1 (Supplementary Figure S3). As seen with the Paclitaxel control, the same effect on membrane disruption and rounding was seen on both MCF-10A and MDA-MB-231 cells when treated with the tubulin polymerization inhibitor vinblastine (Supplementary Figures S3 and S4). In contrast, the microfilament structure composed of actin fibers remained unaltered, indicating that microtubules are the main targets of PTA-1. On the other hand, the DMSO-treated and untreated cells displayed intact microtubules (Figure 6). Thus, these images suggest that PTA-1 disrupts microtubule organization.

### 3.7. PTA-1 Inhibits Tubulin Polymerization

**Figure 7:**
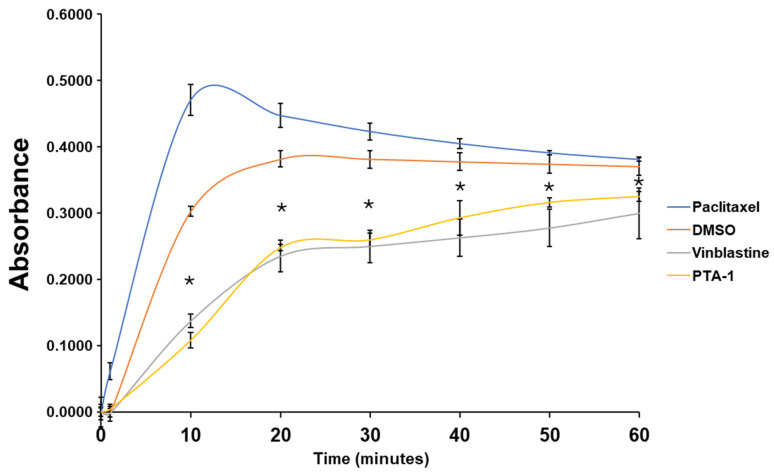
PTA-1 inhibits tubulin polymerization. Curves of four different treatments for tubulin polymerization. Since absorbance is directly correlated with tubulin polymerization, less absorbance indicates less tubulin polymerization. PTA-1 produced significantly less tubulin polymerization than the vehicle control DMSO at every measurement. PTA-1 and the well-known tubulin polymerization inhibitor vinblastine have similar curves. To compare PTA-1 and DMSO, three independent measurements were taken every 10 min. Asterisk (*) represents a statistical difference of * ≤0.000144 when DMSO and PTA-1 were compared.

The ability of PTA-1 to act as a tubulin polymerization inhibitor was directly evaluated using a tubulin polymerization assay kit and a spectrophotometer. MDA-MB-231 cells were treated with PTA-1 (10 µM), DMSO (0.9%) as a vehicle control, Paclitaxel (PTX, 10 µM) as a tubulin stabilizer, and vinblastine (3 µM) as a tubulin polymerization inhibitor. Figure 7 depicts the curves of tubulin polymerization for DMSO, PTX, vinblastine, and the PTA-1 compound. Tubulin polymerization is directly correlated with absorbance, since there is an increase in absorbance at 340 nm when it takes place. As expected, the growth phase of tubulin polymerization after PTX treatment started immediately, with a consistent increment in polymerization during the first 10 min (Figure 7). DMSO, vinblastine, and PTA-1 had a growth phase starting after 4 min, indicating that the nucleation phase lasted 4 min. Interestingly, PTA-1’s polymerization increments were very similar to the ones seen in the well-known tubulin inhibitor vinblastine, with both curves being significantly lower than those observed for DMSO and PTX (Figure 7). These data indicate that PTA-1 acts similarly to vinblastine as a tubulin polymerization inhibitor.

## 4. Discussion

Cancer is still a deadly disease if left untreated, and chemotherapy remains the standard primary cancer treatment. Unfortunately, tumor cells become resistant to many of the anticancer drugs that are currently in use. Therefore, there is an urgent need for new molecules with anticancer properties [47]. In this study, 5600 compounds from the ChemBridge DIVERset chemical library were analyzed to identify cytotoxic compounds. This screen identified a novel compound, 2-{4-[4-methoxy-3-(trifluoromethyl) phenyl]-1H-pyrazol-1-yl}-N-(2-methyl-2H-1,2,3-triazol-4-yl), or PTA-1 (Figure 1), that was subsequently tested on a panel of different cancer cell lines and one non-cancerous cell line. In prior research, we identified two compounds from the same chemical library, P3C [25] and Tpz-1 [19], with significant cytotoxic activity, that have distinct chemical structures as PTA-1. However, our unpublished work has revealed that P3C is a weaker tubulin polymerization inhibitor than PTA-1. Of the 12,240 compounds that have been screened from the same library, only two compounds, PTA-1 and P3C [25], were found to inhibit tubulin polymerization. Thus, the hit rate for tubulin polymerization inhibitors was approximately 1 out of 6000. In subsequent experiments, PTA-1 was tested on blood cancer cell lines for 48 h, whereas cells derived from solid tumors and the non-cancerous cell line were tested for 72 h, due to the higher division rate of blood cancer cells compared to adherent cancer cell lines (Table 1). PTA-1 displayed potent cytotoxic activity in all cancer cell lines examined. Moreover, the least sensitive was a non-cancerous cell line (MCF-10A), which indicates the selectivity of PTA-1 towards cancer cells (Table 1). The triple-negative breast cancer cell line, MDA-MB-231, was used as our model to characterize PTA-1 further. This cell line was used based on our previous experience characterizing other novel compounds [25,48]. Compounds containing a pyrazole moiety have been shown to induce apoptosis [49,50,51]; for this reason, PTA-1 was evaluated to determine the possibility of inducing apoptosis. Our subsequent analyses revealed that PTA-1 induced PS externalization from the inner part of the cell membrane to the exterior (Figure 2A), which is one of the hallmarks of apoptosis. PS externalization is an “eat-me” signal to remove apoptotic cells by phagocytes in vivo [34,52]. To corroborate apoptosis as the cell death mechanism induced by PTA-1, the activation of caspase-3/7 was analyzed. A significant number of cells with activated caspase-3/7 were found after treatment with PTA-1 (Figure 2B). These results confirmed that PTA-1 induces apoptosis as the main cell death mechanism. The induction of apoptosis by small compounds is preferred to necrosis since cells are eliminated through an orderly process [53]. In addition, necrotic cells trigger the activation of the immune system, which leads to the damage of the surrounding tissue [53]. Moreover, apoptosis was further corroborated while analyzing the cell cycle profile. The higher percentages of cells in the sub-G0/G1 (hypodiploid) phase of the cell cycle compared to the vehicle control showed that PTA-1 causes significant DNA fragmentation (Figure 3A), another hallmark of apoptosis. Furthermore, this analysis also showed that PTA-1 arrests the cell cycle in the S and G2/M phases (Figure 3C,D), producing a more dramatic arrest in the G2/M phase (Figure 3D). Compounds with the same target have a similar gene expression signature [54]. Thus, to identify the PTA-1 mode of action, transcriptome analyses were performed to identify the gene expression profile produced by PTA-1 treatment. MDA-MB-231 cells treated with PTA-1 for 24 h showed the upregulation of 198 genes and the downregulation of 532 genes. The Connectivity Map (CMap) database contains a collection of gene profiles of cells that were treated with compounds whose target has been identified [40,55]. To compare the genes affected by PTA-1 treatment, we analyzed 300 DEGs (input size limit) containing the top 150 upregulated and downregulated genes. The results revealed that PTA-1 induced a gene expression profile similar to that of tubulin inhibitors (Figure 4), which indicated that PTA-1 is likely a tubulin inhibitor. Moreover, utilizing molecular docking, the excellent binding potential of PTA-1 with tubulin was found, with a docking score of −12.345 (Figure 5A), which was higher than that of 89U (−10.92), the inhibitor used to crystallize tubulin (Figure 5B). Additionally, PTA-1 showed an MMGBSA score of −73.78 Kcal/Mol, whereas U89 scored −72.89 Kcal/Mol. These in silico analyses predicted that PTA-1 binds strongly to tubulin. Thus far, the CMap analysis has classified PTA-1 as a tubulin inhibitor due to its transcriptional signature, and the molecular modeling illustrated the great possibility of a strong interaction between PTA-1 and tubulin. In an attempt to demonstrate that PTA-1 is a bona fide tubulin inhibitor, we determined that it indeed disrupts the tubular structure of microtubules using fluorescent microscopy. Our results revealed that after 4 h of PTA-1 treatment, the typical tubular structure of tubulin on MDA-MB-231, PANC-1, and A549 cells was completely disrupted, with smeared empty regions that lacked polymerized tubulin (Figure 6 and Supplementary Figures S1 and S2). In addition, a very similar effect on membrane disruption was observed after the treatment of MCF-10A and MDA-MB-231 cells with PTA-1 and vinblastine (see Supplementary Figures S3 and S4). Taken together, these data demonstrate that PTA-1 disorganizes the microtubule structure, which results in the shrinking and rounding of the cell morphologies of the three cancer cell lines analyzed. Lastly, to validate PTA-1 as a tubulin inhibitor, a tubulin polymerization inhibition assay was performed. This assay uses absorbance to test the polymerization of tubulin. Absorbance is directly correlated to tubulin polymerization. The assay revealed that PTA-1 inhibited tubulin polymerization in a manner similar to the well-known tubulin inhibitor vinblastine, a drug currently used for cancer treatment (Figure 7). The absorbance values produced by PTA-1 were significantly lower than those seen with DMSO (Figure 7). Altogether, these results demonstrate that PTA-1 inhibits tubulin polymerization. Microtubules are essential in segregating the chromosomes during mitosis [56,57], which also correlates with tubulin inhibitors arresting the cell cycle in the G2/M phase [58,59]. Cancer cells have an altered cell cycle, which allows them to divide faster. Hence, compounds that stop cell division are considered important anticancer agents. Since known tubulin inhibitors create resistance in cancer cells, PTA-1 could be tested as an alternative to inhibit tubulin polymerization in these cells. These data show that PTA-1 can be considered as a potential chemotherapeutic agent, although in vivo studies should be performed to better understand the effects of PTA-1 in animals.

## Figures and Tables

**Table 1:** Cytotoxic concentration 50% (CC_50_) and selective cytotoxicity index (SCI) values of PTA-1 on a panel of human cell lines.

Cell Line	Tissue Origin	CC_50_ (µM) *	SCI **
Solid Tumor/Adherent Cell Lines		72 h	
MCF-10A	Breast Non-Cancerous	4.40	N/A
MDA-MB-231	Breast Cancer	0.93	4.73
MCF-7	“	0.23	19.13
HCC 70	“	0.74	5.94
T47D	“	0.63	6.98
A375	Melanoma	0.34	12.94
HT-29	Colon Cancer	0.47	9.36
Colo 205	“	0.30	14.66
A549	Lung Cancer	0.17	25.88
HepG2	Liver Cancer	0.36	12.22
PANC-1	Pancreatic Cancer	0.28	1571
PC3	Prostate Cancer	0.92	4.78
Blood Non-Adherent Cell Lines		48 h	
CEM	Lymphomas/Leukemias	0.42	10.47
HL-60	“	0.82	5.36
Ramos	“	0.75	5.86
NALM6	“	2.07	2.12
Jurkat	“	0.32	13.75
KCL22	“	0.72	6.11

* The CC_50_ denotes the cytotoxic concentration of the experimental compound necessary to kill 50% of the cell population after 72 h or 48 h of drug exposure. ** The selective cytotoxicity index (SCI) was calculated by dividing the CC_50_ of non-cancerous MCF-10A cells (used as a reference) by the CC_50_ of the single cancer cell lines. N/A = non-applicable.

## Data Availability

Data are contained within the article and Supplementary Materials.

## Supplementary Materials

The following supporting information can be downloaded at: https://www.mdpi.com/article/10.3390/cells13141225/s1, Figure S1: PTA-1 disturbs microtubule organization in PANC-1 cells.; Figure S2: PTA-1 disturbs microtubule organization in A549 cells; Figure S3: PTA-1 and Vinblastine disrupt tubulin organization in non-cancerous breast MCF-10A cells; Figure S4: PTA-1 and Vinblastine disturb microtubule organization in non-cancerous breast MCF-10A cells; Table S1: 150 upregulated genes in MDA-MB-231 cells after 24 h of treatment with PTA-1 that were analyzed in CMap; Table S2: 150 downregulated genes in MDA-MB-231 cells after 24 h of treatment with PTA-1 that were analyzed in CMap.

## References

[1] What Is Cancer?—National Cancer Institute. https://www.cancer.gov/about-cancer/understanding/what-is-cancer.

[2] Cancer—Symptoms and Causes—Mayo Clinic. https://www.mayoclinic.org/diseases-conditions/cancer/symptoms-causes/syc-20370588.

[3] Hanahan D., Weinberg R.A. (2011). Hallmarks of Cancer: The next Generation. Cell.

[4] Wang B., He F., Hu Y., Wang Q., Wang D., Sha Y., Wu J. (2022). Cancer Incidence and Mortality and Risk Factors in Member Countries of the “ Belt and Road “ Initiative. BMC Cancer.

[5] Siegel R.L., Miller K.D., Wagle N.S., Jemal A. (2023). Cancer Statistics, 2023. CA Cancer J. Clin..

[6] Debela D.T., Muzazu S.G., Heraro K.D., Ndalama M.T., Mesele B.W., Haile D.C., Kitui S.K., Manyazewal T. (2021). New Approaches and Procedures for Cancer Treatment: Current Perspectives. SAGE Open Med..

[7] Sung H., Ferlay J., Siegel R.L., Laversanne M., Soerjomataram I., Jemal A., Bray F. (2021). Global Cancer Statistics 2020: GLOBOCAN Estimates of Incidence and Mortality Worldwide for 36 Cancers in 185 Countries. CA Cancer J. Clin..

[8] Siegel R.L., Giaquinto A.N., Jemal A. (2024). Cancer Statistics, 2024. CA Cancer J. Clin..

[9] Miller K.D., Nogueira L. (2022). Cancer Treatment and Survivorship Statistics, 2022. CA Cancer J. Clin..

[10] Mc Erlain T., Burke A., Branco C.M. (2021). Life after Cell Death-Survival and Survivorship Following Chemotherapy. Cancers.

[11] Anand U., Dey A., Chandel A.K.S., Sanyal R., Mishra A., Pandey D.K., De Falco V., Upadhyay A., Kandimalla R., Chaudhary A. (2023). Cancer Chemotherapy and beyond: Current Status, Drug Candidates, Associated Risks and Progress in Targeted Therapeutics. Genes Dis..

[12] Khachatryan H., Olszowy B., Barrero C.A., Gordon J., Perez-Leal O. (2023). Identification of Inhibitors of Tubulin Polymerization Using a CRISPR-Edited Cell Line with Endogenous Fluorescent Tagging of β-Tubulin and Histone H1. Biomolecules.

[13] Di Cesare E., Verrico A., Miele A., Giubettini M., Rovella P., Coluccia A., Famiglini V., La Regina G., Cundari E., Silvestri R. (2017). Mitotic Cell Death Induction by Targeting the Mitotic Spindle with Tubulin-Inhibitory Indole Derivative Molecules. Oncotarget.

[14] Singh H., Kumar M., Nepali K., Gupta M.K., Saxena A.K., Sharma S., Bedi P.M.S. (2016). Triazole Tethered C5-Curcuminoid-Coumarin Based Molecular Hybrids as Novel Antitubulin Agents: Design, Synthesis, Biological Investigation and Docking Studies. Eur. J. Med. Chem..

[15] Hawash M. (2022). Recent Advances of Tubulin Inhibitors Targeting the Colchicine Binding Site for Cancer Therapy. Biomolecules.

[16] El-Brolsy H.M.E.M., Hanafy N.A.N., El-Kemary M.A. (2022). Fighting Non-Small Lung Cancer Cells Using Optimal Functionalization of Targeted Carbon Quantum Dots Derived from Natural Sources Might Provide Potential Therapeutic and Cancer Bio Image Strategies. Int. J. Mol. Sci..

[17] Lema C., Varela-Ramirez A., Aguilera R.J. (2011). Differential Nuclear Staining Assay for High-Throughput Screening to Identify Cytotoxic Compounds. Curr. Cell. Biochem..

[18] Varela-Ramirez A., Costanzo M., Carrasco Y.P., Pannell K.H., Aguilera R.J. (2011). Cytotoxic Effects of Two Organotin Compounds and Their Mode of Inflicting Cell Death on Four Mammalian Cancer Cells. Cell Biol. Toxicol..

[19] Hess J.D., Macias L.H., Gutierrez D.A., Moran-Santibanez K., Contreras L., Medina S., Villanueva P.J., Kirken R.A., Varela-Ramirez A., Penichet M.L. (2022). Identification of a Unique Cytotoxic Thieno[2,3-c]Pyrazole Derivative with Potent and Selective Anticancer Effects In Vitro. Biology.

[20] Robles-Escajeda E., Das U., Ortega N.M., Parra K., Francia G., Dimmock J.R., Varela-Ramirez A., Aguilera R.J. (2016). A Novel Curcumin-like Dienone Induces Apoptosis in Triple-Negative Breast Cancer Cells. Cell. Oncol..

[21] Robles-Escajeda E., Lerma D., Nyakeriga A.M., Ross J.A., Kirken R.A. (2013). Searching in Mother Nature for Anti-Cancer Activity: Anti-Proliferative and Pro-Apoptotic Effect Elicited by Green Barley on Leukemia/Lymphoma Cells. PLoS ONE.

[22] Abdelhameed R.F.A., Habib E.S., Ibrahim A.K., Yamada K., Abdel-Kader M.S., Ahmed S.A., Ibrahim A.K., Badr J.M., Nafie M.S. (2021). Chemical Constituent Profiling of Phyllostachys Heterocycla Var. Pubescens with Selective Cytotoxic Polar Fraction through EGFR Inhibition in HepG2 Cells. Molecules.

[23] Contreras L., Medina S., Schiaffino Bustamante A.Y., Borrego E.A., Valenzuela C.A., Das U., Karki S.S., Dimmock J.R., Aguilera R.J. (2022). Three Novel Piperidones Exhibit Tumor-Selective Cytotoxicity on Leukemia Cells via Protein Degradation and Stress-Mediated Mechanisms. Pharmacol. Rep..

[24] Martínez-Castillo M., Villegas-Sepúlveda N., Meraz-Rios M.A., Hernández-Zavala A., Berumen J., Coleman M.A., Orozco L., Cordova E.J. (2018). Curcumin Differentially Affects Cell Cycle and Cell Death in Acute and Chronic Myeloid Leukemia Cells. Oncol. Lett..

[25] Gutierrez D.A., Contreras L., Villanueva P.J., Borrego E.A., Morán-Santibañez K., Hess J.D., Dejesus R., Larragoity M., Betancourt A.P., Mohl J.E. (2022). Identification of a Potent Cytotoxic Pyrazole with Anti-Breast Cancer Activity That Alters Multiple Pathways. Cells.

[26] Bolger A.M., Lohse M., Usadel B. (2014). Trimmomatic: A Flexible Trimmer for Illumina Sequence Data. Bioinformatics.

[27] Parrish N., Hormozdiari F., Eskin E. (2014). Assembly of Non-Unique Insertion Content Using next-Generation Sequencing. The Impact of Accurate Quantification on Proteomic and Genetic Analysis and Research.

[28] Langmead B., Salzberg S.L. (2012). Fast Gapped-Read Alignment with Bowtie 2. Nat. Methods.

[29] Love M.I., Huber W., Anders S. (2014). Moderated Estimation of Fold Change and Dispersion for RNA-Seq Data with DESeq2. Genome Biol..

[30] Zhang S., Lyons N., Koedam M., van de Peppel J., van Leeuwen J.P.T.M., van der Eerden B.C.J. (2022). Identification of Small Molecules as Novel Anti-Adipogenic Compounds Based on Connectivity Map. Front. Endocrinol..

[31] Musa A., Ghoraie L.S., Zhang S.-D., Glazko G., Yli-Harja O., Dehmer M., Haibe-Kains B., Emmert-Streib F. (2018). A Review of Connectivity Map and Computational Approaches in Pharmacogenomics. Brief. Bioinform..

[32] Swain R.M., Contreras L., Varela-Ramirez A., Hossain M., Das U., Valenzuela C.A., Penichet M.L., Dimmock J.R., Aguilera R.J. (2022). Two Novel Piperidones Induce Apoptosis and Antiproliferative Effects on Human Prostate and Lymphoma Cancer Cell Lines. Investig. New Drugs.

[33] David T.I., Adelakun N.S., Omotuyi O.I., Metibemu D.S., Ekun O.E., Eniafe G.O., Inyang O.K., Adewumi B., Enejoh O.A., Owolabi R.T. (2018). Molecular Docking Analysis of Phyto-Constituents from Cannabis Sativa with PfDHFR. Bioinformation.

[34] Van Engeland M., Nieland L.J.W., Ramaekers F.C.S., Schutte B., Reutelingsperger C.P.M. (1998). Annexin V-Affinity Assay: A Review on an Apoptosis Detection System Based on Phosphatidylserine Exposure. Cytometry.

[35] Nagata S., Tanaka M. (2017). Programmed Cell Death and the Immune system. Nat. Rev. Immunol..

[36] Hoose S.A., Duran C., Malik I., Eslamfam S., Shasserre S.C., Downing S.S., Hoover E.M., Dowd K.E., Smith R., Polymenis M. (2012). Systematic Analysis of Cell Cycle Effects of Common Drugs Leads to the Discovery of a Suppressive Interaction between Gemfibrozil and Fluoxetine. PLoS ONE.

[37] Kluska M., Woźniak K. (2021). Natural Polyphenols as Modulators of Etoposide Anti-Cancer Activity. Int. J. Mol. Sci..

[38] Iorio F., Bosotti R., Scacheri E., Belcastro V., Mithbaokar P., Ferriero R., Murino L., Tagliaferri R., Brunetti-Pierri N., Isacchi A. (2010). Discovery of Drug Mode of Action and Drug Repositioning from Transcriptional Responses. Proc. Natl. Acad. Sci. USA.

[39] Pabon N.A., Xia Y., Estabrooks S.K., Ye Z., Herbrand A.K., Süß E., Biondi R.M., Assimon V.A., Gestwicki J.E., Brodsky J.L. (2018). Predicting Protein Targets for Drug-like Compounds Using Transcriptomics. PLoS Comput. Biol..

[40] Lim N., Pavlidis P. (2021). Evaluation of Connectivity Map Shows Limited Reproducibility in Drug Repositioning. Sci. Rep..

[41] How Do I Interpret Connectivity Scores, and What Is a “Good” Score?. https://clue.io/connectopedia/connectivity_scores.

[42] Schrödinger What Is Considered a Good GlideScore?. https://www.schrodinger.com/kb/639.

[43] What Is the Difference between the Docking Score and GlideScore from the Results of a Docking Run?|Schrödinger. https://www.schrodinger.com/kb/348.

[44] Schrödinger Can I Relate MM-GBSA Energies to Binding Affinity?. https://www.schrodinger.com/kb/1647.

[45] Stanton R.A., Gernert K.M., Nettles J.H., Aneja R. (2011). ChemInform Abstract: Drugs That Target Dynamic Microtubules: A New Molecular Perspective. ChemInform.

[46] Kapoor S., Srivastava S., Panda D. (2018). Indibulin Dampens Microtubule Dynamics and Produces Synergistic Antiproliferative Effect with Vinblastine in MCF-7 Cells: Implications in Cancer Chemotherapy. Sci. Rep..

[47] Bukowski K., Kciuk M., Kontek R. (2020). Molecular Sciences Mechanisms of Multidrug Resistance in Cancer Chemotherapy. Int. J. Mol. Sci..

[48] Villanueva P.J., Gutierrez D.A., Contreras L., Parra K., Segura-Cabrera A., Varela-Ramirez A., Aguilera R.J. (2021). The Antimalarial Drug Pyronaridine Inhibits Topoisomerase II in Breast Cancer Cells and Hinders Tumor Progression In Vivo HHS Public Access. Clin. Cancer Drugs.

[49] Wang C.R., Wang Z.F., Shi L., Wang Z.C., Zhu H.L. (2018). Design, Synthesis, and Biological Evaluation of Pyrazole Derivatives Containing Acetamide Bond as Potential BRAFV600E Inhibitors. Bioorg. Med. Chem. Lett..

[50] Balbi A., Anzaldi M., MacCi C., Aiello C., Mazzei M., Gangemi R., Castagnola P., Miele M., Rosano C., Viale M. (2011). Synthesis and Biological Evaluation of Novel Pyrazole Derivatives with Anticancer Activity. Eur. J. Med. Chem..

[51] Mellini P., Marrocco B., Borovika D., Polletta L., Carnevale I., Saladini S., Stazi G., Zwergel C., Trapencieris P., Ferretti E. (2018). Pyrazole-Based Inhibitors of Enhancer of Zeste Homologue 2 Induce Apoptosis and Autophagy in Cancer Cells. Philos. Trans. R. Soc. B Biol. Sci..

[52] Birge R.B., Boeltz S., Kumar S., Carlson J., Wanderley J., Calianese D., Barcinski M., Brekken R.A., Huang X., Hutchins J.T. (2016). Phosphatidylserine Is a Global Immunosuppressive Signal in Efferocytosis, Infectious Disease, and Cancer. Cell Death Differ..

[53] Elmore S. (2007). Apoptosis: A Review of Programmed Cell Death. Toxicol. Pathol..

[54] Wang K., Sun J., Zhou S., Wan C., Qin S. (2013). Prediction of Drug-Target Interactions for Drug Repositioning Only Based on Genomic Expression Similarity. PLoS Comput. Biol..

[55] Subramanian A., Narayan R., Corsello S.M., Peck D.D., Natoli T.E., Lu X., Gould J., Davis J.F., Tubelli A.A., Asiedu J.K. (2017). A Next Generation Connectivity Map: L1000 Platform and the First 1,000,000 Profiles. Cell.

[56] Kline-Smith S.L., Walczak C.E. (2004). Mitotic Spindle Assembly and Chromosome Segregation: Refocusing on Microtubule Dynamics. Mol. Cell.

[57] Bunning A.R., Gupta M.L.J. (2023). The Importance of Microtubule-Dependent Tension in Accurate Chromosome Segregation. Front. Cell Dev. Biol..

[58] Salerni B.L., Bates D.J., Albershardt T.C., Lowrey C.H., Eastman A. (2010). Vinblastine Induces Acute, Cell Cycle Phase—Independent Apoptosis in Some Leukemias and Lymphomas and Can Induce Acute Apoptosis in Others When Mcl-1 Is Suppressed. Mol. Cancer Ther..

[59] Thomas E., Gopalakrishnan V., Hegde M., Kumar S., Karki S.S., Raghavan S.C., Choudhary B. (2016). A Novel Resveratrol Based Tubulin Inhibitor Induces Mitotic Arrest and Activates Apoptosis in Cancer Cells. Sci. Rep..

