# Supplementary material for: A Novel Pyrazole Exhibits Potent Anticancer Cytotoxicity via Apoptosis, Cell Cycle Arrest, and the Inhibition of Tubulin Polymerization in Triple-Negative Breast Cancer Cells
Source: Cells. 2024 Jul 20;13(14):1225. doi: 10.3390/cells13141225 (PMC11274517; doi:10.3390/cells13141225)
Supplement: Supplementary file 1 [file cells-13-01225-s001.zip › cells-3066460-supplementary.pdf]

Supplementary Material

A novel pyrazole exhibits selective and potent cytotoxicity *via* apoptosis, S- and G2/M-phase arrest, and tubulin polymerization inhibition in triple-negative breast cancer cells.

Edgar A. Borrego [1,2,*], Cristina D. Guerena[1,2,3], Austre Y. Schiaffino Bustamante[1,2,4], Denisse A. Gutierrez[1,2], Carlos A. Valenzuela[1,2,5], Ana P. Betancourt[1,2], Armando Varela-Ramirez[1,2], Renato J. Aguilera[1,2,*]

[1] The Border Biomedical Research Center, the University of Texas El Paso, El Paso, TX 79968, United States.

[2] Department of Biological Sciences, the University of Texas El Paso, TX 79968, United States.

[3] Current address: the UT Southwestern Medical Center, Dallas, TX 75390, United States.

[4] Current address: Cancer Biology Program, Graduate Division of Biological and Biomedical Sciences, Emory University, Atlanta, GA 30322, United States.

[5] Current address: Department of Oncology Science, University of Oklahoma Health Sciences Center, Oklahoma City, OK 73104.

*Corresponding authors: EAB- eaborregopu@miners.utep.edu; RJA- raguilera@utep.edu

Table of Contents

Supplementary Material

1. ***Supplementary Table S1.*** **150 Upregulated genes in MDA-MB-231 cells after 24 hours of treatment with PTA-1 that were input in CMap**
2. ***Supplementary Table S2.*** **150 Downregulated genes in MDA-MB-231 cells after 24 hours of treatment with PTA-1 that were input in CMap**
3. ***Supplementary Figure S1.*** **PTA-1 and Placlitaxel disturb microtubule organization in pancreatic carcinoma PANC-1 cells.**
4. ***Supplementary Figure S2.*** **PTA-1 and Paclitaxel disturb microtubule organization in lung carcinoma epithelial A549 cells.**
5. ***Supplementary Figure S3.*** **Vinblastine disturbs microtubule organization in triple-negative breast MDA-MB231 cells.**
6. ***Supplementary Figure S4.*** **PTA-1 and Vinblastine disturb microtubule organization in non-cancerous breast MCF-10A cells.**

Supplementary Table S1. 150 Upregulated genes in MDA-MB-231 cells after 24 hours of treatment with PTA-1 that were input in CMap.

HAS2	SMURF2	LAMA1	ADGRF5	CENPE	NAV3
UBASH3B	PLAUR	APBB1IP	GFRA1	HHIP	ANOS1
LCP1	S1PR1	CALD1	ZNF804A	TNFSF15	SEMA7A
ADAMTS1	EVA1A	NAV2	ARHGAP22	CASC8	CREB5
GLIPR1	FLNC	AKAP2	ST6GALNAC5	IL1A	CALB2
CORO2B	UCA1	ALS2CL	IGFBP1	NIPAL1	SH3RF2
MARCH4	WNT5B	SMAGP	SAMD12	GCOM1	AKAP12
RGMB	PEAR1	CHRNB1	SLC45A3	GPC5	ANTXR2
NEDD4L	KRTAP2-3	LOC152225	PTPRR	TMC7	CAB39
ADAMTS6	DUSP8	KHDRBS3	RELN	LINC00704	FAM196B
UAP1	TAGLN	SPHK1	NLRP3	IL6R	ATP8B1
MGLL	HAS2-AS1	FRMD5	PDZD2	MGAM	SLC28A3
SOCS2	FGF5	SLCO1B3	KIF18A	LANCL3	PITPNC1
TPM4	SLC20A2	ITGA6	AURKA	TMEM217	AOC2
CORO1C	LRRC8C	HDAC9	TMEM71	KRT34	GEM
HBEGF	EPB41L4B	PLAT	CENPA	RPSAP52	GAS2L3
PROSER2	GALNT6	CPED1	RGMB-AS1	WNK4	CD177
ANKRD13A	IL24	LINC00707	ARID3A	MPP4	SERPINB2
RNF145	FHL2	SERF1A	COL17A1	EN2	SERPINA9
SPOCD1	C1orf110	CD274	TSPAN12	DDR2	MMP3
SSR3	ETS1	NFATC2	KISS1	RGPD2	TM4SF1
NEXN	ARHGAP29	KCNQ5	DAW1	KIF17	SMTN
TMEM171	PLXNA2	ODC1	ITGB3	AIM1L	SERPINE1
SH3BP5L	AEN	BMPER	BORA	SUSD4	RAB8B
FGF1	AOX1	VCAN	LRRN4	DDIT4L	CNTN1

Supplementary Table S2. 150 Downregulated genes in MDA-MB-231 cells after 24 hours of treatment with PTA-1 that were input in CMap.

**IL6**	**PDE7B**	**CRISPLD2**	**ARHGEF40**	**ZNF608**	**ID1**
**LOC284454**	**ZFP36**	**FHDC1**	**PARP14**	**RASSF5**	**DDIT4**
**BHLHE40**	**NOL4L**	**HERC6**	**PLEKHG4**	**PADI4**	**NFKBIA**
**IFIT2**	**NNMT**	**EGLN3**	**SDC2**	**DBP**	**IGFN1**
**IFIT1**	**PBX1**	**PARD6B**	**SYT12**	**DDX60**	**PLEKHS1**
**TNFAIP2**	**CSF1**	**ELFN2**	**NFIL3**	**FOXQ1**	**PFKFB4**
**IFIT3**	**DUSP6**	**SORBS2**	**PLSCR4**	**NREP**	**WT1**
**TSC22D3**	**LFNG**	**SMAD7**	**TUB**	**PPFIA4**	**ICOSLG**
**TRIM16L**	**OASL**	**FPR1**	**COL16A1**	**COL7A1**	**DUSP1**
**ADAMTS15**	**NEDD9**	**FAM20C**	**RSRP1**	**RRAD**	**WNT7B**
**IRF1**	**ASAP3**	**SSBP2**	**C1QTNF6**	**VAMP1**	**CX3CL1**
**TNFSF10**	**TRIB2**	**SCNN1A**	**GABRE**	**BMF**	**GYG2**
**PDE4B**	**KLF9**	**SPACA6P**	**CMKLR1**	**PLA2G6**	**LOC440300**
**FOSB**	**ID3**	**CYP1B1**	**MIR210HG**	**PPL**	**ABCC3**
**FOS**	**IKBKE**	**PDGFRB**	**JUNB**	**CEBPD**	**SPRY1**
**PTGES**	**XAF1**	**NR4A2**	**GBP4**	**APOL1**	**NR4A1**
**PGF**	**ARRDC3**	**SLC2A3**	**ALDH3B1**	**PIK3C2B**	**CXXC5**
**MAP3K14**	**ZC3H12A**	**KIAA1217**	**ATP1B1**	**PTPN6**	**EPHB3**
**SCD**	**BCL3**	**IFI44**	**PTGS2**	**MAP3K1**	**CDH11**
**ATF3**	**SLC1A3**	**CITED2**	**BTN3A3**	**CSGALNACT1**	**CALHM2**
**CIITA**	**BTN3A1**	**SECTM1**	**EDN2**	**DHX58**	**SMAD6**
**BMP4**	**MYO18A**	**VDR**	**NEAT1**	**IER5L**	**METTL7A**
**RBM47**	**ELF3**	**APOL6**	**IRF2BPL**	**SAA2**	**NR1H3**
**ATOH8**	**IFIH1**	**LAMA5**	**FZD1**	**PIR**	**SGK223**
**TUBA1A**	**RNF43**	**SAMD9L**	**PADI1**	**RNF207**	**RARRES3**

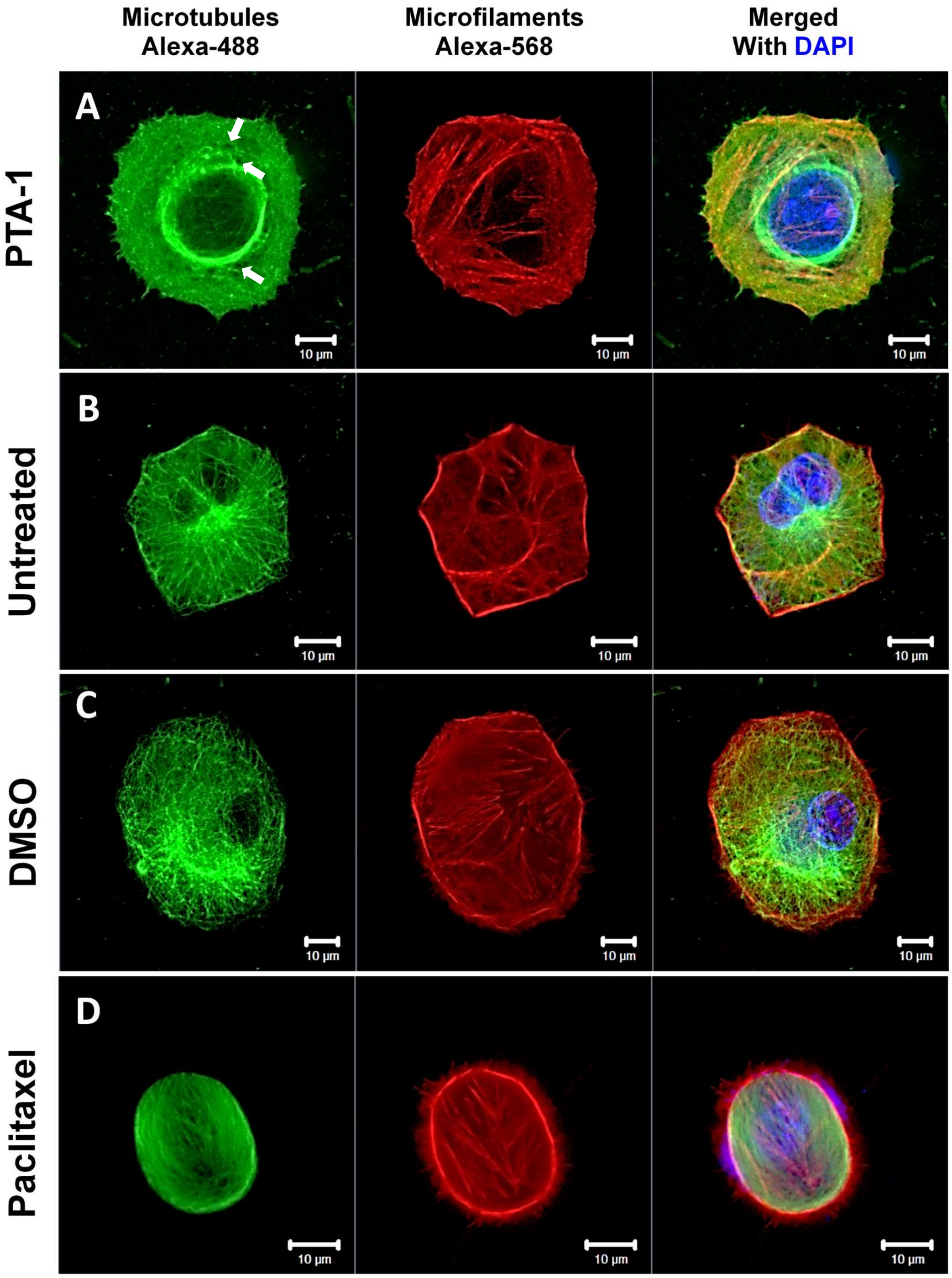

Supplementary Figure S1. **PTA-1 disturbs microtubule organization in PANC-1 cells.** Cells were treated with 20 µM of PTA-1 for 4 h. Next, PANC-1 cells were stained with anti-α-tubulin antibody conjugated to Alexa-488 (microtubules), phalloidin-Alexa-568 (microfilaments, polymerized actin), and DAPI (nucleus) and analyzed via confocal microscopy. **A** PTA-1 treatment disrupted the microtubule organization. White arrows indicate empty regions where tubulin is absent. **B** Untreated **C** DMSO (vehicle), and **D** paclitaxel (microtubule stabilizing agent).

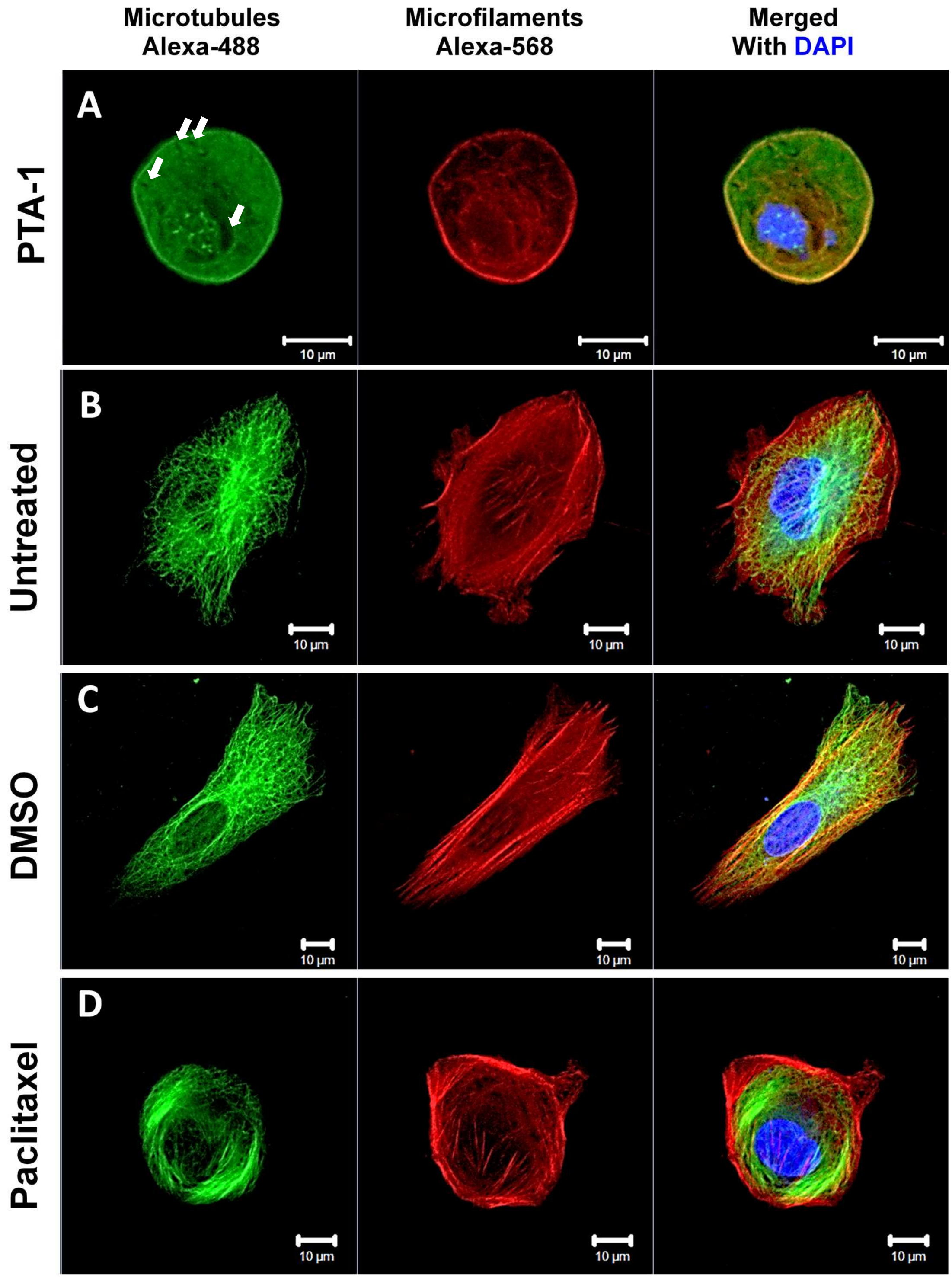

Supplementary Figure S2. **PTA-1 disturbs microtubule organization in cells.** A549 cells were treated with **A** PTA-1 **B** Untreated **C** DMSO (vehicle) and **D** paclitaxel (microtubule stabilizing agent). A549 cells were treated for 4 h and then fixed and stained as detailed in the Material and Methods section. White arrows indicate empty regions where tubulin is absent.

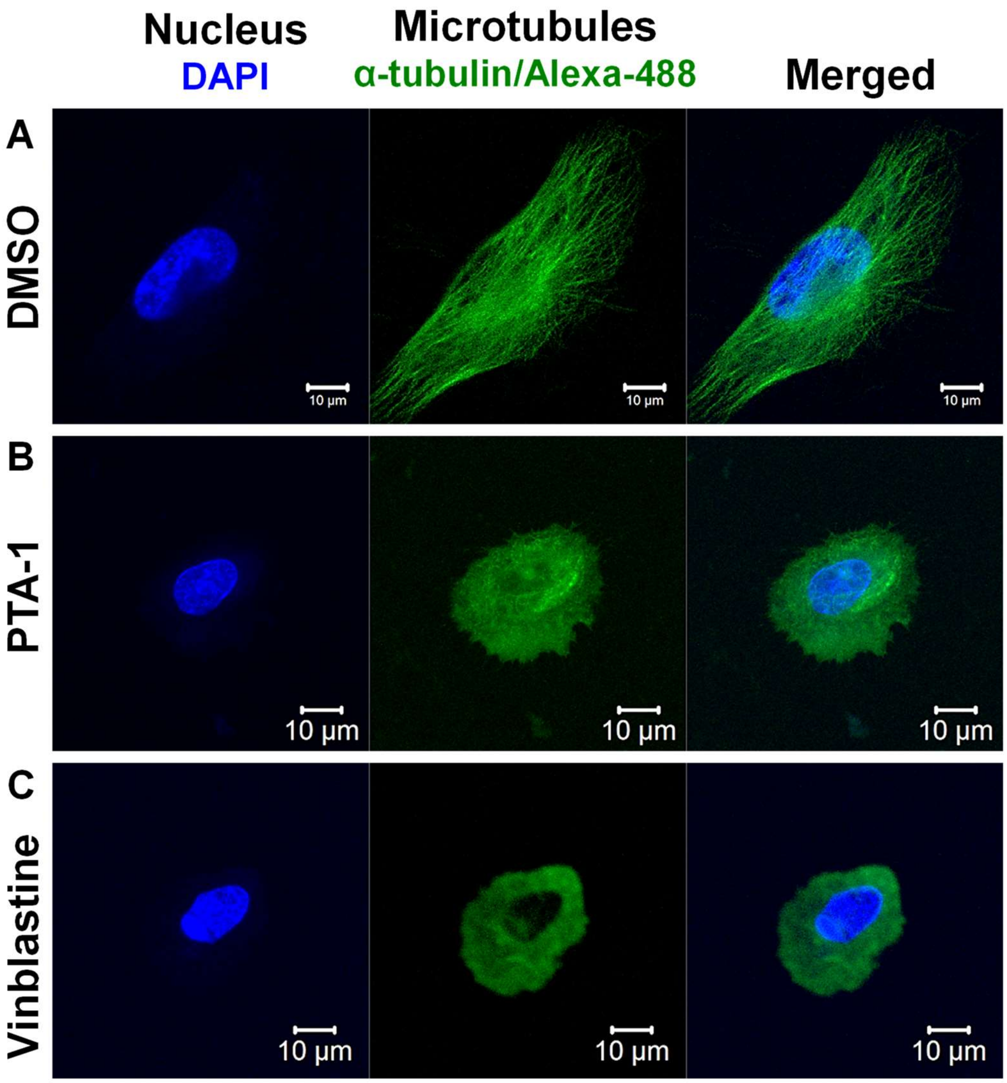

Supplementary Figure S3. PTA-1 and Vinblastine disrupt tubulin organization in non-cancerous breast MCF-10A cells. After 4 h of exposure to PTA-1 (**B**, 20 µM), Vinblastine (**C**, 3 µM) and DMSO (**A**, vehicle control) cells were fixed, permeabilized, and stained with DAPI (nucleus) and α-tubulin/Alexa-488 (microtubules) and analyzed *via* confocal microscopy subsequently. Vinblastine (**C**) was included as a positive control for tubulin polymerization inhibition.

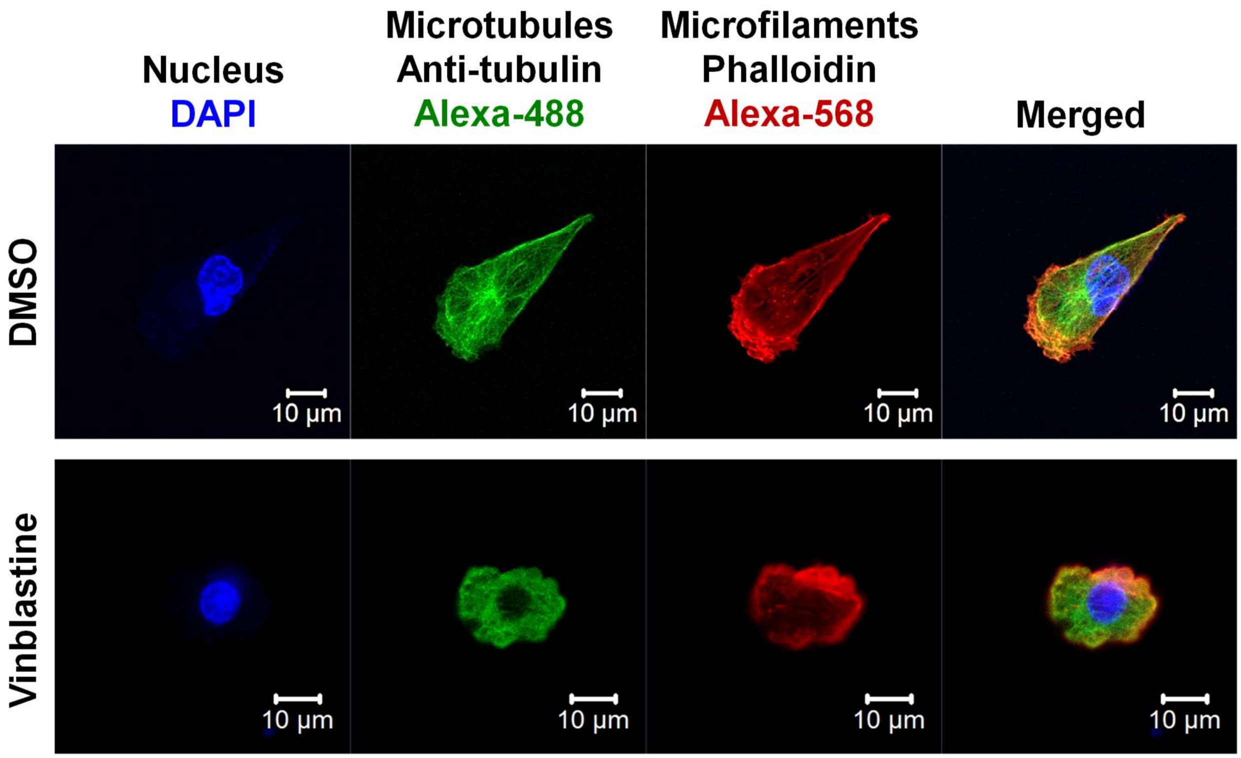

Supplementary Figure S4**.** Vinblastine disrupts microtubule organization in Triple-negative breast MDA-MB-231 cells analyzed *via* confocal microscopy; DMSO (vehicle control) is also included. After treatment, cells were stained with DAPI (nucleus), anti-tubulin/ Alexa-488 (microtubules), and Phalloidin/Alexa-568 (microfilaments).
